# Supplementary material for: Keratoconus-susceptibility gene identification by corneal thickness genome-wide association study and artificial intelligence IBM Watson
Source: Commun Biol. 2020 Jul 31;3:410. doi: 10.1038/s42003-020-01137-3 (PMC7395727; doi:10.1038/s42003-020-01137-3)
Supplement: Supplementary file 1 — Supplementary Information [file 42003_2020_1137_MOESM1_ESM.pdf]

Supplementary Information for

Keratoconus-susceptibility genes identification by corneal thickness genome-wide association study and artificial intelligence IBM Watson

***Corresponding Author:**

Masahiro Miyake, MD, PhD, MPH

Department of Ophthalmology and Visual Sciences

Kyoto University Graduate School of Medicine

54 Shogoin, Kawahara, Sakyo, Kyoto 606-8507, Japan

Tel.: +81-75-751-3248

Fax: +81-75-752-0933

This PDF file includes:

Supplementary Figures 1

Supplementary Table 1 to 4

Supplementary Note

SI References

Supplementary Figure 1: Quantile–quantile (QQ) plot from the discovery stage.

A QQ plot of the associations between all analyzed SNPs and myopic maculopathy in the discovery stage. Each blue dot represents an observed *P*-value (represented on a $-\log_{10}$ scale) versus the corresponding expected *P*-value before genomic control, whereas each red dot represents the observed *P*-value versus the corresponding expected *P*-value after genomic control. The black line corresponds to the null distribution.

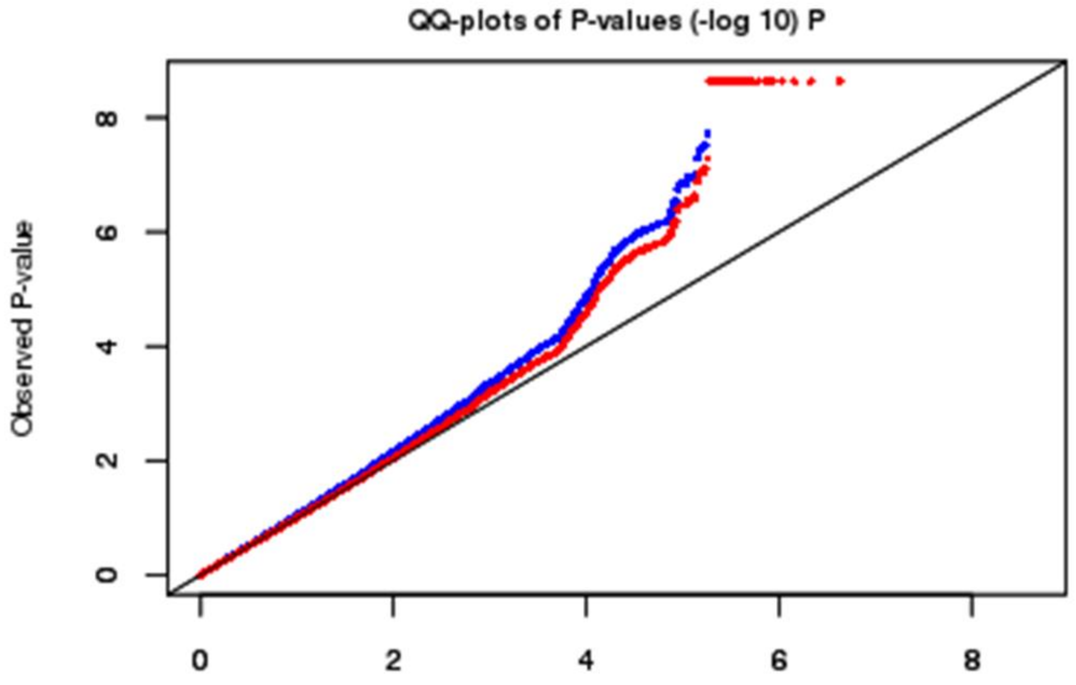

Supplementary Table 1. Description of the Nagahama cohort for our two-stage genome-wide association study (GWAS) of corneal thickness (CCT)

Stage	N	Female (%)	Age (years)	CCT (μm)	AL (mm)
Discovery	3,584	2,445 (68.2)	56.4 ± 13.0	544 ± 28	24.08 ± 1.34
Replication	2,942	2,000 (68.0)	58.6 ± 12.2	545 ± 28	23.98 ± 1.33

AL, average axial length in both eyes. The data shown are expressed as the mean ± standard deviation.

Supplementary Table 2. List of candidate genes included in the Watson predictive analysis

Gene	Comment	Included in Watson analysis?
LOC100132571	No literature	No
LOC101926919	No literature	No
LOC101927874	No literature	No
LOC285419	No literature	No
BC045810	No literature	No
LOC100506700	No literature	No
LOC100419872	No literature	No
LOC400456	No literature	No
STON2	Teacher gene	No
FNDC3B	Teacher gene	No
FOXO1	Teacher gene	No
NRXN1		Yes
SPTBN1		Yes
MEIS1		Yes
GLS		Yes
ARNT		Yes
OSBPL10		Yes
SLC7A11		Yes
CPLX2		Yes
SUPT3H		Yes
CSMD1		Yes
ADRA1A		Yes
FAM49B		Yes
C10orf68		Yes
PLEKHS1		Yes
ADAM12		Yes
MGMT		Yes
MTCH2		Yes
PKNOX2		Yes
ADAMTS15		Yes
KCNA6		Yes
SLCO1B1		Yes
CIT		Yes
DOCK9		Yes

SLC24A4	Yes
SMAD3	Yes
SEMA4B	Yes
CIB1	Yes
TTC23	Yes
ADAMTS17	Yes
CERS3	Yes
WWOX	Yes
CDH13	Yes
SMG6	Yes
GAS7	Yes
SLC47A2	Yes
ALDH3A1	Yes
DCAKD	Yes
NMT1	Yes
TBX4	Yes
ALPK2	Yes
ARHGEF1	Yes
MKKS	Yes

Supplementary Table 3. Similarity scores of 42 genes analysed using Watson for Drug Discovery (WDD)

Gene	Similarity score	Rank
SMAD3	0.062	1
CDH13	0.05	2
GAS7	0.049	3
CIB1	0.046	4
MEIS1	0.046	5
ARNT	0.043	6
DOCK9	0.04	7
CSMD1	0.038	8
WWOX	0.037	9
NRXN1	0.036	10
SPTBN1	0.036	11
ADAM12	0.035	12
PLEKHS1	0.034	13
ADAMTS17	0.027	14
ADAMTS15	0.027	15
CPLX2	0.024	16
SEMA4B	0.024	17
ARHGEF1	0.024	18
MGMT	0.024	19
TTC23	0.024	20
OSBPL10	0.023	21
MTCH2	0.023	22
SUPT3H	0.023	23
MKKS	0.023	24
CERS3	0.023	25
ALDH3A1	0.023	26
SMG6	0.022	27
SLC7A11	0.022	28
GLS	0.022	29
NMT1	0.022	30
ADRA1A	0.022	31

DCAKD	0.015	32
PKNOX2	0.013	33
FAM49B	0.013	34
SLC24A4	0.013	35
ALPK2	0.013	36
SLC47A2	0.013	37
SLCO1B1	0.012	38
CIT	0.004	39
TBX4	0.004	40
KCNA6	0.003	41
C10ORF68	0.003	42

Supplementary Table 4. Description of the Asian cohorts included in CCT meta analysis

Study	N	Female (%)	Age (years)	CCT (μm)	AL (mm)	Genotyping platform	Imputation	CCT-measurement method
Nagahama (discovery)	3,584	2,445 (68.2)	56.4 ± 13.0	544 ± 28	24.08 ± 1.34	HumanHap610 Quad, HumanOmni2.5, CoreExome24, and HumanExome	MACH	TX-20P
Nagahama (replication)	2,942	2,000 (68.0)	58.6 ± 12.2	545 ± 28	23.98 ± 1.33	TaqMan SNP genotyping assay	-	TX-20P
SiMES	2,510	1,244 (49.6)	59.6 ± 11.0	540 ± 33	-	HumanHap610 Quad	Minimac	Ultrasonic pachymetry
SCES	1,861	952 (51.2)	58.5 ± 9.5	553 ± 33	-	HumanHap610 Quad	Minimac	Ultrasonic pachymetry
SCES2	608	312 (51.3)	60.4 ± 9.5	552 ± 33	-	OmniExpress	Minimac	Ultrasonic pachymetry
SINDI	2,508	1,288 (51.4)	58.0 ± 10.0	540 ± 33	-	HumanHap610 Quad	Minimac	Ultrasonic pachymetry

The data shown are expressed as the mean ± standard deviation.

Supplementary Notes

SiMES, SCES, and SINDI cohorts

Each cohort was comprised of a population-based, cross-sectional group of Malay, Chinese, and Indian adults, aged 40 – 80 years. The details of the cohort designs were described previously.[1–3] Briefly, an age-stratified random sampling of adults residing in Singapore, aged 40 – 80 years, was drawn from a computer-generated random list of names provided by the Ministry of Home Affairs, for each cohort. A final sampling frame of residents was derived from this list using an age-stratified random sampling strategy.

BeadChip DNA arrays, namely the OmniExpress chip and HumanHap610 Quad chip (Illumina, San Diego, CA, US), were used to determine the sample genotypes. The *STON2* rs2371597 genotypes were determined by genotype imputation. Imputation was performed using Minimac software (https://genome.sph.umich.edu/wiki/Minimac).

Keratoconus cohort

Japanese patients with keratoconus (n = 179) were recruited from the Yokohama City University Hospital. All procedures adhered to the tenets of the Declaration of Helsinki. The Institutional Review Board and the Ethics Committee of each participating institute approved the study protocols. All patients were fully informed of the purpose and procedures of the study, and written consent was received from each patient prior to their participation in the study. All patients underwent a comprehensive ophthalmic

examination, including corneal topography and visual acuity evaluation. Keratoconus was diagnosed as an eye with corneal thinning, corneal scaring, or significant visual acuity loss.

A BeadChip DNA array, namely the Human OmniExpress chip (Illumina), was used to determine the genotypes of patients with keratoconus. The genotypes of *CPLX2* rs4242187, *ADAM12* rs11244890, *SMAD3* rs12913547, and *CDH13* rs1035533 were directly determined using the chip, and the genotypes of *STON2* rs2371597, *NRXN1* rs13382330, *CSMD1* rs143428993, and *WWOX* rs6564538 were determined by genotype imputation with the 1000 Genomes dataset (phase 3, v5 release) as a reference panel.

Japanese control cohorts

Control subjects from Yokohama City University

Normal Japanese subjects (N = 1018) were recruited from the Yokohama City University Hospital. A BeadChip DNA array, namely the Human OmniExpress chip (Illumina) was used to determine the genotypes of patients with keratoconus. Samples with a call rate of <97% were excluded. SNPs were excluded, based on the following quality-control criteria: a call rate of <98%, significantly different rates of missing data between patients and control subjects ($P < 1.0 \times 10^{-6}$), an overall minor allele frequency of <1%, and a significant deviation from Hardy–Weinberg equilibrium (HWE) in the control subjects ($P < 1.0 \times 10^{-5}$). Additionally, cryptic relatedness between samples was estimated based on identity by descent; closely related samples with a pi-hat > 0.1875 were eliminated. The Michigan imputation server (https://imputationserver.sph.umich.edu/index.html#!pages/home) was used, with the

1000 Genomes dataset (phase3 v5 release) serving as a reference panel. All imputed SNPs were filtered using the following quality-control parameters: a MAF of >0.01 and a squared correlation between imputed and true genotypes (r^2) of >0.7. The genotypes of *CPLX2* rs4242187, *ADAM12* rs7089454, *SMAD3* rs12913547, and *CDH13* rs1035533 were directly determined using the chip, and the genotypes of *STON2* rs2371597, *NRXN1* rs13382330, *CSMD1* rs143428993, and *WWOX* rs6564538 were determined by genotype imputation with the 1000 Genomes dataset (phase 3, v5 release) as a reference panel.

Nagahama cohort

A detailed description of the Nagahama cohort is provided in the Online Methods section.

ToMMo database

The Integrative Japanese Genome Variation Database (version 3.5KJPN, https://ijgvd.megabank.tohoku.ac.jp/) provides genomic reference panels obtained from 3,554 normal Japanese subjects. The details of the cohort were described previously.[4–6] Briefly, samples were recruited from the Tohoku Medical Megabank Organization, Iwate Medical Megabank Organization, Nagahama Prospective Cohort for Comprehensive Human Bioscience, and National Hospital Organization Nagasaki Medical Center. The whole-genome sequences of all DNA samples were obtained using an Illumina HiSeq 2500 instrument. The resulting dataset contains the allele-frequency data of 37,067,715 reliable autosomal SNVs detected by whole-genome sequencing of 3,552 Japanese individuals (3.5KJPN, released September 28, 2017). We used a dataset

comprised of 7,931,579 SNVs with allele frequencies of ≥1% in the Japanese population.

References

1. Cornes, B. K. *et al.* Identification of four novel variants that influence central corneal thickness in multi-ethnic Asian populations. *Hum. Mol. Genet.* **21,** 437–445 (2012).
2. Lavanya, R. *et al.* Methodology of the Singapore Indian Chinese Cohort (SICC) eye study: quantifying ethnic variations in the epidemiology of eye diseases in Asians. *Ophthalmic Epidemiol.* **16,** 325–336 (2009).
3. Foong, A. W. P. *et al.* Rationale and methodology for a population-based study of eye diseases in Malay people: the Singapore Malay Eye Study (SiMES). *Ophthalmic Epidemiol.* **14,** 25–35 (2007).
4. Kuriyama, S. *et al.* The Tohoku Medical Megabank Project: design and mission. *J. Epidemiol.* **26,** 493–511 (2016).
5. Nagasaki, M. *et al.* Rare variant discovery by deep whole-genome sequencing of 1,070 Japanese individuals. *Nat. Commun.* **6,** 8018 (2015).
6. Yamaguchi-Kabata, Y. *et al.* iJGVD: an integrative Japanese genome variation database based on whole-genome sequencing. *Hum. Genome Var.* **2,** 15050

(2015).
