# Supplementary material for: Keratoconus-susceptibility gene identification by corneal thickness genome-wide association study and artificial intelligence IBM Watson
Source: Commun Biol. 2020 Jul 31;3:410. doi: 10.1038/s42003-020-01137-3 (PMC7395727; doi:10.1038/s42003-020-01137-3)
Supplement: Supplementary file 4 — Description of Additional Supplementary Files [file 42003_2020_1137_MOESM4_ESM.pdf]

Description of Additional Supplementary Files

File Name: **Supplementary Data 1**
Description: All SNPs with P value > 1.0×10-4 in the discovery GWAS.

File Name: **Supplementary Data 2**
Description: Associations between previously reported genes with CCT in the GWAS discovery stage
