# Supplementary material for: Keratoconus-susceptibility gene identification by corneal thickness genome-wide association study and artificial intelligence IBM Watson
Source: Commun Biol. 2020 Jul 31;3:410. doi: 10.1038/s42003-020-01137-3 (PMC7395727; doi:10.1038/s42003-020-01137-3)
Supplement: Supplementary file 5 — Reporting Summary [file 42003_2020_1137_MOESM5_ESM.pdf]

nature research

Corresponding author(s): Masahiro Miyake
Last updated by author(s): Jul 1, 2020

Reporting Summary

Nature Research wishes to improve the reproducibility of the work that we publish. This form provides structure for consistency and transparency in reporting. For further information on Nature Research policies, see Authors & Referees and the Editorial Policy Checklist.

Statistics

For all statistical analyses, confirm that the following items are present in the figure legend, table legend, main text, or Methods section.

n/a	Confirmed	
☐	☒	The exact sample size (n) for each experimental group/condition, given as a discrete number and unit of measurement
☐	☒	A statement on whether measurements were taken from distinct samples or whether the same sample was measured repeatedly
☐	☒	The statistical test(s) used AND whether they are one- or two-sided<br>*Only common tests should be described solely by name; describe more complex techniques in the Methods section.*
☐	☒	A description of all covariates tested
☐	☒	A description of any assumptions or corrections, such as tests of normality and adjustment for multiple comparisons
☐	☒	A full description of the statistical parameters including central tendency (e.g. means) or other basic estimates (e.g. regression coefficient) AND variation (e.g. standard deviation) or associated estimates of uncertainty (e.g. confidence intervals)
☐	☒	For null hypothesis testing, the test statistic (e.g. F, t, r) with confidence intervals, effect sizes, degrees of freedom and P value noted<br>*Give P values as exact values whenever suitable.*
☐	☒	For Bayesian analysis, information on the choice of priors and Markov chain Monte Carlo settings
☐	☒	For hierarchical and complex designs, identification of the appropriate level for tests and full reporting of outcomes
☐	☒	Estimates of effect sizes (e.g. Cohen's d, Pearson's r), indicating how they were calculated

Our web collection on statistics for biologists contains articles on many of the points above.

Software and code

Policy information about availability of computer code

Data collection	No software was used for data collection.
Data analysis	R ver 3.5.2, PLINK ver. 1.9 and Watson Drug Discovery were used for data analysis

For manuscripts utilizing custom algorithms or software that are central to the research but not yet described in published literature, software must be made available to editors/reviewers. We strongly encourage code deposition in a community repository (e.g. GitHub). See the Nature Research guidelines for submitting code & software for further information.

Data

Policy information about availability of data

All manuscripts must include a data availability statement. This statement should provide the following information, where applicable:

- Accession codes, unique identifiers, or web links for publicly available datasets
- A list of figures that have associated raw data
- A description of any restrictions on data availability

The complete GWAS summary data can be visualised at figshare (https://figshare.com/articles/dataset/CCT/12592529). The datasets generated during the current study are also available from the corresponding author on reasonable request.

Field-specific reporting

Please select the one below that is the best fit for your research. If you are not sure, read the appropriate sections before making your selection.

☒ Life sciences ☐ Behavioural & social sciences ☐ Ecological, evolutionary & environmental sciences

Life sciences study design

All studies must disclose on these points even when the disclosure is negative.

Sample size	For the two-staged GWAS, data from 8,289 healthy Japanese volunteers (34 – 80 years of age) without physical impairment enrolled in the Nagahama Prospective Cohort for Comprehensive Human Bioscience (the Nagahama Study) was used.<br>To investigate the association between identified gene and keratoconus, we performed a case-control analysis using data from 179 patients with keratoconus from Yokohama City University and pooled the data of 9,589 Japanese healthy control subjects.<br><br>We revealed that this keratoconus assocoiation analysis has 94% statistical power, indicating the sufficient power to demonstrate the presence of an association (CaTS software, http://csg.sph.umich.edu//abecasis/CaTS/index.html. Following values were used to estimate the power; Cases=180, controls= 9589, significant level = 0.05, Disease Model = Multiplicative, prevalence = 0.0005, Disease Allele Frequency = 0.3, Genotype Relative Risk = 1.5).
Data exclusions	The dataset of patients were fixed before analysis, and no data were excluded.
Replication	Four stage replication analysis from four independent cohort consist of Japanese, Malay, Chinese and Indian were performed. In all stages, the associations of STON2 rs2371597 with central corneal thickness (CCT) were consistently replicated, and meta-analysis strengthened the effect.
Randomization	Three principal components were used as covariates in the discovery GWAS, and we got an acceptable control; the genomic inflation factor lambda (λGC) was 1.065).
Blinding	All participants underwent ophthalmic examinations, including objective determinations of the CCT (TX-20P; Canon, Tokyo, Japan) and axial lengths (IOLMaster; Carl Zeiss Meditec, Inc., Dublin, CA, USA).<br><br>Diagnosis of keratoconus and genotyping were independently performed;<br>Initially, ophthalmologists diagnosed and recruited keratoconus patients who visited Yokohama City University without any genotype information. All patients who were diagnosed as keratoconus were genotyped and analyzed.

Reporting for specific materials, systems and methods

We require information from authors about some types of materials, experimental systems and methods used in many studies. Here, indicate whether each material, system or method listed is relevant to your study. If you are not sure if a list item applies to your research, read the appropriate section before selecting a response.

Materials & experimental systems

n/a	Involved in the study
☒	☐ Antibodies
☒	☐ Eukaryotic cell lines
☒	☐ Palaeontology
☐	☒ Animals and other organisms
☐	☒ Human research participants
☒	☐ Clinical data

Methods

n/a	Involved in the study
☒	☐ ChIP-seq
☒	☐ Flow cytometry
☒	☐ MRI-based neuroimaging

Animals and other organisms

Policy information about studies involving animals; ARRIVE guidelines recommended for reporting animal research

Laboratory animals	Adult C57BL/6 mice.
Wild animals	The study involve no wild animals.
Field-collected samples	The study did not involve samples collected from the field.
Ethics oversight	The Kyoto University Graduate School and Faculty of Medicine Ethics Committee approved the study protocol and the procedures

Note that full information on the approval of the study protocol must also be provided in the manuscript.

Human research participants

Policy information about studies involving human research participants

Population characteristics	In the discovery GWAS: 2,445 (68.2%) of 3,584 samples were female and the mean age of participants was 56.4 years. The mean CCT was 544 μm and the mean axial length was 24.08 mm. In the replication stage using rest of samples from Nagahama cohort: the mean age was 58.6 years, and 2,942 of them were female. The mean CCT was 545 μm and the mean axial length was 23.98 mm.
Recruitment	For the two-staged GWAS, we analysed data from healthy Japanese volunteers enrolled in the Nagahama Prospective Cohort for Comprehensive Human Bioscience (the Nagahama Study). We used the first follow-up data collected from 8,289 participants between 2013 and 2015 from the general population of Nagahama City, Japan. Community residents (34 – 80 years of age) without physical impairment were eligible for the second stage of the Nagahama Study.<br><br>In the trans-ethnic replication stages, we included three Asian cohorts, namely the SiMES, SCES, and SINDI cohorts, which consisted of Malay (n = 2,510), Chinese (n = 2,469), and Indian (n = 2,508) subjects, respectively.<br><br>We performed a case-control analysis using data from 179 patients with keratoconus from Yokohama City University and pooled the data of 9,589 Japanese healthy control subjects from Yokohama City University, Nagahama Cohort and Tohoku Medical Megabank Project.
Ethics oversight	The Kyoto University Graduate School and Faculty of Medicine Ethics Committee and the Nagahama Municipal Review Board of Personal Information Protection approved the study protocol and the procedures used to obtain informed consent. All study procedures adhered to the tenets of the Declaration of Helsinki. All participants were fully informed of the purpose and procedures of the study, and written consent was obtained from each participant. Patient records and information were anonymized prior to analysis.

Note that full information on the approval of the study protocol must also be provided in the manuscript.
